# *CD1* Gene Polymorphisms and Phenotypic Variability in X-Linked Adrenoleukodystrophy

**DOI:** 10.1371/journal.pone.0029872

**Published:** 2012-01-12

**Authors:** Mathieu Barbier, Audrey Sabbagh, Edwige Kasper, Muriel Asheuer, Ornella Ahouansou, Ingrid Pribill, Sonja Forss-Petter, Michel Vidaud, Johannes Berger, Patrick Aubourg

**Affiliations:** 1 UMR 745, Faculté des Sciences Pharmaceutiques et Biologiques, Université Paris Descartes, Sorbonne Paris Cité, Paris, France; 2 Center for Brain Research, Medical University of Vienna, Vienna, Austria; 3 UMR 216, Institut de Recherche pour le Développement (IRD), Paris, France; 4 Laboratoire de Parasitologie, Faculté des Sciences Pharmaceutiques et Biologiques, Université Paris Descartes, Sorbonne Paris Cité, Paris, France; UCL Institute of Neurology, United Kingdom

## Abstract

X-linked adrenoleukodystrophy (X-ALD) is characterized by marked phenotypic variation ranging from adrenomyeloneuropathy (AMN) to childhood cerebral ALD (CCALD). X-ALD is caused by mutations in the *ABCD1* gene, but no genotype-phenotype correlation has been established so far and modifier gene variants are suspected to modulate phenotypes. Specific classes of lipids, enriched in very long-chain fatty acids that accumulate in plasma and tissues from X-ALD patients are suspected to be involved in the neuroinflammatory process of CCALD. CD1 proteins are lipid- antigen presenting molecules encoded by five *CD1* genes in human (*CD1A-E*). Association studies with 23 tag SNPs covering the *CD1* locus was performed in 52 patients with AMN and 87 patients with CCALD. The minor allele of rs973742 located 4-kb downstream from *CD1D* was significantly more frequent in AMN patients (χ^2^ = 7.6; *P* = 0.006). However, this association was no longer significant after Bonferroni correction for multiple testing. The other polymorphisms of the *CD1* locus did not reveal significant association. Further analysis of other *CD1D* polymorphisms did not detect stronger association with X-ALD phenotypes. Although the association with rs973742 warrants further investigations, these results indicate that the genetic variants of *CD1* genes do not contribute markedly to the phenotypic variance of X-ALD.

## Introduction

X-linked adrenoleukodystrophy (X-ALD; MIM# 300100) is a progressive neurodegenerative disease that is caused by mutations in the *ABCD1* gene, encoding ALD protein (ALDP), an (ATP)-binding-cassette (ABC) transporter located in the peroxisomal membrane [1]. Based on cross complementation in yeast, it is suggested that ALDP is active as a homodimer and is involved in the transport of very long-chain fatty (VLCF) acyl-CoA esters across the peroxisomal membrane [2]. ALDP deficiency results in increased levels of VLCFacyl-CoA esters in the cytosol and consequently all X-ALD patients accumulate saturated straight very-long-chain fatty acids (VLCFA) in their plasma and tissues [3]. The disease affects the cerebral white matter, axons in the spinal cord, peripheral nerves, adrenal cortex and testis [4]. X-ALD is characterized clinically by a striking and unpredictable variation of neurologic phenotype, ranging from the rapidly progressive childhood cerebral form (CCALD) to adrenomyeloneuropathy (AMN) that affects only the spinal cord in adults. Analysis of the *ABCD1* gene in X-ALD patients has revealed a wide variability of *ABCD1* mutations, with virtually one different mutation per family, without correlation with the few recurrent mutations and X-ALD phenotype (http://www.x-ald.nl) [4], [5]. Moreover, as X-ALD males in the same family may express different phenotypes, it is clear that mutations of the *ABCD1* gene does not account for the disease variability. No correlation between X-ALD phenotype and levels of VLCFA accumulation observed in plasma and fibroblasts from X-ALD patients has been demonstrated either.

Patients with AMN develop progressive spastic paraparesis in adulthood, sometimes associated with peripheral nerve involvement. The neuropathology of AMN is mostly characterized by axonal loss in the long tracts of the spinal cord, mainly dorsal fascicles and pyramidal tracts, without significant inflammatory changes [6]. By contrast, boys with CCALD develop a devastating neurologic disease that leads to vegetative stage or death in few years [6]. The neuropathology of CCALD is characterized by severe degeneration of the white matter within the brain, loss of oligodendrocytes, reactive astrocytosis and secondary axonal loss. A most striking change of CCALD is the presence of inflammatory infiltrates at the border of the lesions of the white matter, just behind the active demyelinating front. Inflammatory cells are CD4^+^ and CD8^+^ T lymphocytes, some of them containing granzyme B, and rare B lymphocytes [7].

The phenotypic variation of X-ALD is likely caused by the combination of environmental and/or genetic factors. Genetic segregation analysis provided support for the hypothesis that at least one autosomal gene plays a role in the clinical variation of X-ALD [8]. The mechanisms that lead to neuroinflammation in patients with CCALD are unknown but it has been speculated that specific classes of lipids enriched in VLCFA could be involved this process [9]. In addition, CD1b-immunoreactive astrocytes and strongly CD1c-immunoreactive perivascular and scattered interstitial cells, presumably microglia, are frequently observed in the demyelinating lesions of patients with CCALD [7]. CD1b and CD1c belong to a group of antigen-presenting molecules that can present lipid antigens to immunocompetent T cells [10]. CD1d molecules present glycolipid antigens to a specialized subset of T cells known as natural killer T (NKT) cells [10]. Type I NKT cells (also known as invariant or semi-invariant NKT, iNKT cells) express an invariant T-cell receptor (TCR) α-chain in combination with certain Vα chains. This TCR permits iNKT cells to recognize both self and microbial glycolipid antigens in the context of CD1d molecules [10]. Converging data support the role of iNKT cells in the inflammatory cerebral demyelinating diseases [11]. In humans, five genes (*CD1A-E*) located within 200-kb on chromosome 1q22-q23 encodes CD1a, CD1b, CD1c, CD1d and CD1e lipid antigen-presenting molecules. Polymorphic variants of one or several of these CD1 molecules could affect lipid presentation and, in the context of impaired metabolism of VLCFA as observed in X-ALD, may contribute to trigger the neuroinflammation process. To test this hypothesis, we assessed the association of variants in the *CD1* genes cluster encompassing the five human *CD1* genes in a population of 139 X-ALD patients presenting either CCALD or pure form of AMN.

## Results

### Tagging of the CD1 locus and association analyses of selected tag SNPs in CCALD and AMN patients

We first analyzed the LD pattern in a region of 400-kb centered on *CD1* genes. We observed that the five *CD1* genes were included in a single block of linkage disequilibrium (LD), thus excluding LD of *CD1* genes polymorphisms with polymorphisms of surrounding genes (data not shown). We then focused on the 200-kb region encompassing the five *CD1* genes and selected 23 tag SNPs to cover the common genetic variation of the *CD1* genes cluster.

**Figure 1:**
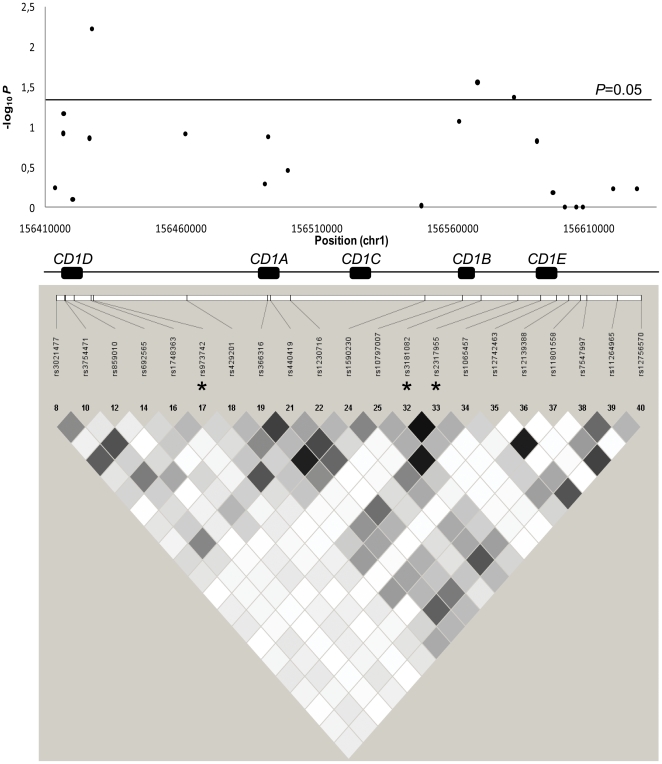
Tagging of the *CD1 locus*. A) Allelic association results of the tag SNPs genotyped in CCALD and AMN patients: each black dot represents a tag SNP; -log_10_ *P* is plotted for each of the 21 tag SNPs; the five *CD1* genes are indicated by black boxes; B) LD between the corresponding tag SNPs: LD is represented by shades of grey as a function of r^2^ values (black diamond for r^2^≥0.90, white diamond for r^2^ = 0). Associated tag SNPs are marked with an asterisk.

A non-synonymous SNP of *CD1A* (rs2269715, p.Cys68Trp) and a SNP located 3-kb upstream *CD1C* (rs12033535) were excluded from further analyses because their minor allele frequencies (MAFs) were <0.05. The remaining 21 SNPs were analyzed in AMN and CCALD patients (Table 1). Only three tag SNPs showed evidence of allelic associations with X-ALD phenotypes at a 0.05 significance level (Table 1): rs973742 G/A (χ^2^ = 7.6; *P* = 0.006; empirical *P* = 0.008) located 4-kb downstream from the 3′ region of *CD1D*; rs3181082 C/T (χ^2^ = 5.3; *P* = 0.028; empirical *P* = 0.031) and rs2317955 G/T (χ^2^ = 4.4; *P* = 0.043; empirical *P* = 0.054) which are in LD (r^2^ = 0.96), and located in the upstream region of *CD1B* (at 1- and 14-kb respectively). For rs973742, rs3181082 and rs2317955, the minor alleles were more frequent in AMN than CCALD patients (Table 1). For rs973742, both the asymptotic (*P* = 0.006) and empirical *P* value (*P* = 0.008) are closed from the threshold required to keep a type I error rate at 0.05 after Bonferroni correction (Bonferroni-corrected significance threshold = 0.004, see Materials and Methods). As rs973742 and rs3181082 are located in the *CD1D* and *CD1B* gene region, respectively, we analyzed the LD pattern of the 21 tag SNPs included in the association analysis (Figure 1). There was no LD between the most associated tag SNPs located in *CDID* and *CD1B* (rs973742 and rs3181082, r^2^ = 0).

**Table 1:** Allelic analyses of the 23 tag SNPs genotyped in CCALD and AMN patients.

SNP (dbSNP31)	MAF	MAF AMN	MAF CCALD	χ^2^	*P* value	empirical *P* value^b^	Odds ratio [CI95%]
rs3021477	0.39	0.41	0.38	0.32	0.573	-	0.87 [0.53 ; 1.42]
rs3754471	0.49	0.43	0.53	2.4	0.121	-	1.47 [0.90 ; 2.40]
rs859010	0.08	0.13	0.06	3.91	0.069^a^	0.064	0.42 [0.18 ; 1.01]
rs692565	0.41	0.42	0.41	0.06	0.805	-	0.94 [0.57 . 1.54]
rs1748363	0.15	0.19	0.13	2.2	0.138	-	0.61 [0.31 ; 1.18]
rs973742	0.40	0.50	0.33	7.56	0.006	0.008	0.50 [0.30 ; 0.82]
rs429201	0.16	0.20	0.13	2.38	0.123	-	0.60 [0.32 ; 1.15]
rs366316	0.16	0.17	0.14	0.43	0.512	-	0.80 [0.41 ; 1.55]
rs2269715	0.04	0.07	0.02	-	-	-	-
rs440419	0.19	0.24	0.16	2.26	0.133	-	0.63 [0.35 ; 1.15]
rs1230716	0.41	0.44	0.39	0.88	0.347	-	0.79 [0.48 ; 1.29]
rs12033535	0.03	0.03	0.03	-	-	-	-
rs1590230	0.16	0.16	0.16	0.003	0.956	-	0.98 [0.51 ; 1.90]
rs10797007	0.28	0.34	0.24	2.94	0.086	0.117	0.62 [0.67 ; 1.07]
rs3181082	0.11	0.16	0.07	5.33	0.028^a^	0.031	0.41 [0.19 ; 0.89]
rs2317955	0.10	0.15	0.08	4.36	0.043^a^	0.054	0.44 [0.20 ; 0.97]
rs1065457	0.28	0.33	0.25	2.07	0.15	-	0.68 [0.40 ; 1.15]
rs12742463	0.08	0.10	0.08	0.39	0.654^a^	-	0.76 [0.32 ; 1.80]
rs12139388	0.08	0.09	0.08	0.03	1^a^	-	0.92 [0.39 ; 2.22]
rs11801558	0.08	0.08	0.08	0.005	1^a^	-	0.97 [0.39 ; 2.42]
rs7547997	0.11	0.11	0.11	0.06	1^a^	-	1.10 [0.50 ; 2.39]
rs11264965	0.18	0.19	0.17	0.29	0.587	-	0.84 [0.45 ; 1.58]
rs12756570	0.06	0.06	0.05	0.29	0.589	-	0.76 [0.27 ; 2.09]

a : The Fisher's exact test was used for SNP with a Minor Allele Frequency (MAF)<0.10.

b : Permutation-based empirical *P* value were calculated for SNP showing a trend of association (*P* value<0.10).

### Screening of sequence variations in CD1D and CD1B genes in controls

*CD1D* and *CD1B* genes, including the 5′ and 3′UTRs, were then sequenced in 50 control individuals to identify potential new variants. All SNPs identified were already described and referenced in dbSNP database (http://www.ncbi.nlm.nih.gov/projects/SNP/). The MAF of detected variants is indicated in Table S1.

### Association analyses of CD1D and CD1B variants in CCALD and AMN patients

Ten additional common SNPs with a MAF>0.05 (four for the *CD1D* gene and six for the *CD1B* gene) were further genotyped in AMN and CCALD patients. Despite a low MAF (<0.05), three SNPs (rs11583390, rs35841099 and rs62642468) of the *CD1B* gene were kept in this study given their potential functionality (missense variants or located in the 3′ UTR).

All variants detected in our control population were also detected in the X-ALD population. These included in particular the two missense variants of *CD1B* but both of them had a low MAF (≤0.03), precluding any possibility to reach significance level of association in our small population of X-ALD patients. No significant association was detected with any of the six SNPs of *CD1B* (Table 2).

**Table 2:** Allelic analyses of *CD1D* and *CD1B* variants in the CCALD and AMN patients.

SNP (dbSNP31)	*gene*	MAF	MAF AMN	MAF CCALD	χ^2^	*P* value	empirical *P* value^b^	Odds ratio [CI95%]
rs859008	*CD1D*	0.09	0.14	0.06	5.99	0.018^a^	0.020	0.36 [0.16 ; 0.84]
rs859009		0.09	0.15	0.06	7.13	0.010^a^	0.010	0.34 [0.15 ; 0.77]
rs859013		0.09	0.14	0.05	6.9	0.014^a^	0.016	0.33 [0.14 ; 0.78]
rs422236		0.41	0.42	0.40	0.12	0.73	-	0.92 [0.56 ; 1.50]
rs11583390	*CD1B*	0.03	0.03	0.03	-	-	-	-
rs16840096		0.11	0.16	0.09	3.22	0.073	0.118	0.51 [0.24 ; 1.08]
rs3176842		0.17	0.17	0.16	0.05	0.82	-	0.93 [0.49 ; 1.78]
rs35841099		0.004	0.01	0	-	-	-	-
rs962879		0.12	0.17	0.09	3.64	0.056	0.106	0.49 [0.23 ; 1.31]
rs62642468		0.03	0.05	0.02	-	-	-	-

a : The Fisher's exact test was used for SNP with a Minor Allele Frequency (MAF)<0.10.

b : Permutation-based empirical *P* value were calculated for SNP showing a trend of association (*P* value<0.10).

In the *CD1D* gene, three intronic SNPs with a MAF∼0.10 were associated with X-ALD phenotypes at the 0.05 significance level (Table 2). For all of them, the minor allele was more frequent in the AMN sample population (rs859008 C/T, *P*_Fisher_ = 0.018, empirical *P* = 0.020; rs859009 C/G, *P*_Fisher_ = 0.010, empirical *P* = 0.010; rs859013 A/G, *P*_Fisher_ = 0.014, empirical *P* = 0.016). These three SNPs were in strong LD (r^2^≥0.87).

Using HapMap (http://hapmap.ncbi.nlm.nih.gov) genotypic data from European individuals, we detected a strong LD between three SNPs of *CD1D* (rs859008, rs859009 and rs859013) and seven SNPs located 3.2-kb upstream from the transcription start site of *CD1D* (r^2^≥0.90). This raises the possibility that the previous associations of rs859008, rs859009 and rs859013 with X-ALD phenotypes resulted from a LD with a possible causal SNP localized in this region. The seven SNPs in the upstream region of *CD1D* formed a 1.3-kb block of strong LD (r^2^≥0.93). BLAST tool (http://blast.ncbi.nlm.nih.gov/Blast.cgi) and the ENSEMBL genome browser (http://www.ensembl.org/Homo_sapiens/Info/Index) revealed that this 1.3-kb region overlaps with a known pseudogene (ENSEMBL identifier: ENSG00000227295). We therefore genotyped these seven SNPs in patients with AMN and CCALD. All SNPs were detected in the X-ALD population, with a similar MAF (<0.10). The seven SNPs also formed a block of total LD in the X-ALD population (r^2^ = 1, Figure S1) and were also in LD with rs859008, rs859009 and rs859013 in X-ALD patients (r^2^≥0.74, Figure S1). Consequently, these seven SNPs showed associations with X-ALD phenotypes, but the significance levels were lower than with rs859008, rs859009 and rs859013 (Table S2).

## Discussion

Besides major histocompatibility complex (MHC) class I and II molecules, a third lineage of antigen-presenting molecules that bind lipid and glycolipid antigens rather than peptides is mediated by the family of CD1 proteins [10]. The molecular basis for the phenotypic variability of X-ALD is still unclear. However, given the putative role of lipid antigens in the development of cerebral demyelinating forms of X-ALD that are characterized by severe neuroinflammation [6], we wondered whether a polymorphism in one or several *CD1* genes may influence the incidence of inflammatory demyelination in X-ALD. We “tagged” the entire region including the five *CD1* genes in 139 X-ALD patients presenting either CCALD or pure AMN with 23 selected tag SNPs identified using the HapMap SNP database. Only one common SNP (rs973742 A/G; MAF = 0.40 in the X-ALD population) located in the downstream region of *CD1D* showed evidence of an allelic association with X-ALD phenotypes. The minor allele A had a higher prevalence in AMN than in CCALD patients 0.50 *vs* 0.33 respectively; χ^2^ = 7.6 *P* = 0.006 Odds ratio = 0.50). Three other intronic SNPs (rs859008, rs859009 and rs859013) of *CD1D* gene showed weaker associations (*P* value ranging from 0.010 to 0.018) with X-ALD phenotypes. These three SNPs are in strong LD with a block of seven SNPs overlapping a known pseudogene in the upstream region of *CD1D*, but we did not detect stronger association between any of these seven SNPs and X-ALD phenotypes.

Re-sequencing of the *CD1D* as well as *CD1B* genes (in which additional SNPs showed weaker allelic association with X-ALD phenotype) in 50 control individuals failed to detect new SNPs/genetic variants that were not already identified in the European HapMap population. One may wonder whether we missed a SNP in the X-ALD population that would not be described in SNP databases and not detected in the healthy control population screened. Our results clearly argue against this hypothesis. First, we did not detect any new SNP in the healthy control population compared to those described in SNP databases. Second, the minor allele frequencies of detected variants in the control population were very similar to those found in SNP databases and in the X-ALD population (Table S1). Third, the pattern of LD between the SNPs of *CD1D* and its 5′ region was the same in the European HapMap population and in the X-ALD population. All together, these data are in favor of a similar pattern of genetic variations in the *CD1* locus between the European HapMap population, the control population, and the X-ALD population. It is therefore unlikely that a frequent and non described SNP existing only in the CCALD or AMN populations has been missed, although we cannot exclude the possibility that rare variants in *CD1D* and *CD1B* may exist in CCALD and AMN patients since our approach did not allow the detection of such variants. Our failure to detect new SNPs confirms the low level of allelic polymorphism in the *CD1* genes [12]. This is in line with the fact that, in contrast to the MHC class I and class II proteins, the CD1 antigen presenting system, which has diverged early in vertebrate evolution from MHC antigen presenting molecules, is a simpler system with a character that has evolved little from the primordial antigen presenting function [13].

The functional role of rs973742 is unknown. CD1d molecules are believed to present glycolipid antigens to a distinct lineage of T cells called iNKT cells, but the exact nature of the endogenous ligand(s) recognized by these cells remains elusive [14]. Data obtained in experimental autoimmune encephalomyelitis (EAE) mice support the crucial role of iNKT cells in the pathogenesis of inflammatory demyelinating diseases [11]. Patients with relapsing multiple sclerosis have smaller numbers of circulating iNKT cells than healthy controls and their function is linked to the severity of the disease [11]. Recently, we showed that the frequency of iNKT cells in peripheral blood mononuclear cells from X-ALD patients was significantly lower than in healthy individuals [15]. X-ALD iNKT cells are also more immature and CD1d expression is decreased in X-ALD B lymphocytes. Cytokine production by X-ALD iNKT cells is however normal and no correlation was observed between iNKT cell maturation or iNKT cell frequency and X-ALD phenotypes. The association between the *CD1D* genetic variant (rs973742) and X-ALD phenotype may warrant further studies on the CD1d molecule itself, in particular on how this variant may affect CD1d recycling between the plasma membrane and the intracellular compartment [13]. Furthermore, we cannot exclude the possibility that rs973742 may be in linkage disequilibrium with a causative variant at distance from the *CD1* locus or with an undetected rare variant of *CD1D* present only in the ALD population. Although our results do not suggest that genetic variants of *CD1* genes contribute markedly to the phenotypic variance of X-ALD, CD1d and other CD1 molecules may still have an important role in the inflammatory process observed in CCALD.

In the absence of biomarkers correlated with X-ALD phenotypes, the identification of genetic variants contributing to the phenotypic variance of X-ALD remains a huge task, as in nearly all rare Mendelian diseases. Using a candidate gene strategy, which has major limitations, no genetic variants have been found to influence significantly X-ALD phenotypes up to now [16], [17]. The results reported herein for the *CD1* genes do not differ significantly from previous studies in this field. Although CCALD and AMN phenotypes can be clearly distinguished at the neuropathological level, the failure to detect genetic variants having a strong influence on these two X-ALD phenotypes could be due to the sum of many rare genetic variant interactions, as observed in many multifactorial diseases [18].

## Materials and Methods

### Patients and healthy volunteers

The population of X-ALD cases included 52 patients with “pure” AMN and 87 patients with childhood cerebral ALD (CCALD), all of European origin (Caucasians). Patients with “pure” AMN are adult males >45 years of age with isolated spinal cord disease with or without adrenal insufficiency but without cerebral demyelinating lesions at brain MRI. Longitudinal follow-up of 684 patients with X-ALD indicates that the risk to develop cerebral demyelinating lesions drop to less than 5% after the age of 45 years (P. Aubourg, unpublished results). Patients with CCALD are boys who developed inflammatory cerebral demyelinating disease before 12 years of age. Thus, two groups of X-ALD patients with extreme phenotypes (CCALD and pure AMN) were defined for this study. DNAs from 50 unrelated healthy volunteers of European origin were used as “control” to screen for new sequence variations in the *CD1D* and *CD1B* genes by sequence analyses. The DNA sample collection was declared to INSERM and approved by the local ethical committee (Comité de Protection des Personnes Ile-de-France III). Informed written consents were obtained from all X-ALD and control patients or their legal guardians.

### Tagging analyses of the CD1 locus and linkage disequilibrium (LD) studies

The pattern of LD across the *CD1* genes cluster was studied in a 400-kb large region encompassing the five *CD1* genes and neighboring genes. All X-ALD cases being of European origin, we used HapMap SNP data (the International HapMap Project (http://hapmap.ncbi.nlm.nih.gov)/Public Release #22) from the CEU HapMap sample (European ancestry) to examine LD block structure with the program Haploview (http://www.broadinstitute.org/haploview) [19]. The r^2^ for all pairs of SNPs was calculated to evaluate the presence of LD between the different *CD1* genes, and between *CD1* genes with non-*CD1* genes. After excluding LD between polymorphisms of *CD1* genes and neighboring genes (data not shown), we used the Tagger program implemented in Haploview to select SNPs that efficiently tagged all common variations in the 200-kb region encompassing only the five *CD1* genes. We chose an aggressive tagging procedure to select tag SNPs, requiring a minimum Minor Allele Frequency (MAF) of 0.05 and a minimum estimated r^2^ between the tagged and tag SNPs sets to be ≥0.90 that allowed only a slight loss of power in typing only tag SNPs. From the original set of 115 common SNPs in the European sample (MAF>0.05), 23 tag SNPs were selected (Table 1). The mean r^2^ between tagged and the tag SNPs set was 0.995.

### Identification of variants in CD1D and CD1B genes

*CD1D* and *CD1B* genes were sequenced in 50 unrelated controls. Two regions, each covering one of these two genes + 1-kb in untranslated regions (UTRs) were targeted for a Polymerase-Chain-Reaction (PCR) amplification using the following primers: for *CD1D*, 5′-GCCTGGCCTCTGTTGATAAACAAA-3′ and 5′-CAGGGTCTCCCTCTGTCATCCTTA-3′; for *CD1B*, 5′-GAGGCAGTAACAGACTGCCAG-3′ and 5′-GCCAAGGGTGGTTTTCTCCAAAG-3′. PCRs were carried out using the Expand Long Range PCR kit (Roche Applied Science, Switzerland), and purified before the sequence analyses (Multiscreen HTS plates, Millipore, USA) following manufacturer's instructions. Primers used for sequencing are available upon request. Sequencing reactions were carried out using the BigDye® Terminator v1.1 kit (Applied Biosystems, USA). Base calling was realized on the ABI 377 sequencer (Applied Biosystems, USA). All polymorphisms were confirmed using double sense sequencing.

### Genotyping of polymorphisms in the X-ALD sample population

TaqMan® SNP Genotyping Assay-by-Design method (Applied Biosystems, USA) was used to genotype 39 of 40 SNPs (23 tag SNPs, nine SNPs in *CD1D/CD1B* genes, seven SNPs in the upstream region of *CD1D*) in the X-ALD sample population with an allele-specific hybridization approach. Pre-designed and Custom probes were designed by Applied Biosystems (available upon request). PCR amplification was carried out according to the manufacturer's instructions. The PCR was followed by allelic discrimination using the ABI Prism 7900 to perform plate reading. Automated allele calling was performed by allelic discrimination plots with ABI SDS software version 2.2 (Applied Biosystems, USA).

The SNP rs859009 was genotyped using the PCR-Restriction-Fragment-Length Polymorphisms (RFLP) method, as this polymorphism creates a restriction site for the restriction enzyme AvrII. The sequence containing the SNP rs859009 was first amplified using the following primers: forward, 5′-AAAAGAGTGAGGGAGAGGGAGGTG-3′ and reverse, 5′-CAACGTGTGGGACGCTTTACAAC-3′. After digestion of PCR products with AvrII (Ozyme, France) and 2% agarose gel (Euromedex, France) electrophoresis, two bands of 457-bp and 251-bp can be identified when the CC genotype is present; one band of 708-bp for the GG genotype; three bands for heterozygous CG. Profiles of digestion were checked by a sequencing of PCR products from six patients. For all SNPs, the genotyping rate was ≥97%. No deviation of the Hardy-Weinberg equilibrium was detected using a significance threshold of 0.05 for all tested SNPs.

### Statistical analysis

We used the PLINK software to carry out statistical analyses (http://pngu.mgh.harvard.edu/purcell/plink/) [20]. For the association analyses, the statistical significance of differences in allele frequencies between AMN and CCALD patients was evaluated using an allelic chi-square test (χ^2^ Pearson), with degree of freedom equal to one. For SNPs with MAF<0.10 in the X-ALD sample, an exact *P*-value was computed using the Fisher's exact test. To take into account the small size of the X-ALD sample population, and to deal with rare variants, an empirical *P*-value was computed using an adaptive permutation procedure for SNPs which showed trends (*P*<0.10) or evidences (*P*<0.05) of associations. Since some of the tag SNPs were in LD in the X-ALD sample population (Figure 1), accounting for the number of tests using a simple Bonferroni correction is expected to be too much conservative. An effective number of independent SNPs was therefore calculated by the method proposed by Li and Ji [21] implemented in the SNPSpD software [22]. We found that the 21 tag SNPs included in statistical analyses (exclusion of two tag SNPs with MAF<0.05) corresponded to 12 effective independent ones. The corresponding Bonferroni-corrected significance threshold was 0.004.

## Supporting Information

Figure S1 **LD between genotyped SNPs of** ***CD1D*** **and its upstream region in X-ALD patients.** Structure of *CD1D* is indicated by an arrow for the transcription start site and black boxes for exons; LD is represented by shades of grey as a function of r^2^ values (black square for r^2^≥0.90, white diamond for r^2^ = 0). Previously associated SNPs are marked with an asterisk. The black line underlines the seven SNPs (1–7), which form a block of LD.
(DOC)

Table S1 **Screening of common variants of** ***CD1D*** **and** ***CD1B*** **genes in controls.**
(DOC)

Table S2 **Analyses of variants in the upstream region of** ***CD1D*** **in CCALD and AMN patients.**
(DOC)
