# Supplementary material for: CD1 Gene Polymorphisms and Phenotypic Variability in X-Linked Adrenoleukodystrophy
Source: PLoS One. 2012 Jan 12;7(1):e29872. doi: 10.1371/journal.pone.0029872 (PMC3257241; doi:10.1371/journal.pone.0029872)
Supplement: Figure S1 — LD between genotyped SNPs of CD1D and its upstream region in X-ALD patients. Structure of CD1D is indicated by an arrow for the transcription start site and black boxes for exons; LD is represented by shades of grey as a function of r2 values (black square for r2≥0.90, white diamond for r2 = 0). Previously associated SNPs are marked with an asterisk. The black line underlines the seven SNPs (1–7), which form a block of LD. (DOC) [file pone.0029872.s001.doc]

**Supplementary informations**

**Figure S1**. **LD between genotyped SNPs of *CD1D* and its upstream region in X-ALD patients**. Structure of *CD1D* is indicated by an arrow for the transcription start site and black boxes for exons; LD is represented by shades of grey as a function of r² values (black square for r² ≥ 0.90, white diamond for r² = 0). Previously associated SNPs are marked with an asterisk. The black line underlines the seven SNPs (1-7), which form a block of LD.

**Figure S1**.
