# Supplementary material for: CD1 Gene Polymorphisms and Phenotypic Variability in X-Linked Adrenoleukodystrophy
Source: PLoS One. 2012 Jan 12;7(1):e29872. doi: 10.1371/journal.pone.0029872 (PMC3257241; doi:10.1371/journal.pone.0029872)
Supplement: Table S1 — Screening of common variants of CD1D and CD1B genes in controls. (DOC) [file pone.0029872.s002.doc]

**Supplementary informations**

**Table S1**. Screening of common variants of *CD1D* and *CD1B* genes in controls.

| SNP (dbSNP131) | position (NCBI36/hg18) | MAF ^a^ | MAF ^b^ | MAF^c^ |
| --- | --- | --- | --- | --- |
| rs859008 | Chr1:156416680 | 0.11 | 0.12 | 0.09 |
| rs859009 | Chr1:156416752 | 0.11 | 0.13 | 0.09 |
| rs859013 | Chr1:156417842 | 0.11 | 0.13 | 0.09 |
| rs422236 | Chr1:156421717 | 0.46 | 0.43 | 0.41 |
| rs62642468 | Chr1:156567228 | 0.01 | NA | 0.03 |
| rs962879 | Chr1:156566930 | 0.16 | 0.14 | 0.12 |
| rs35841099 | Chr1:156566464 | 0.004 | NA | 0.004 |
| rs3176842 | Chr1:156564981 | 0.24 | 0.16 | 0.17 |
| rs16840096 | Chr1:156564584 | 0.18 | 0.14 | 0.11 |
| rs11583390 | Chr1:156564488 | 0.07 | 0.07 | 0.03 |

NA: unavailable.

^a^ Minor allele frequency in the control population.

^b^ Minor allele frequency from HapMap CEU European data (Release 28).

^c^ Minor allele frequency in X-ALD population.
