# Supplementary material for: CD1 Gene Polymorphisms and Phenotypic Variability in X-Linked Adrenoleukodystrophy
Source: PLoS One. 2012 Jan 12;7(1):e29872. doi: 10.1371/journal.pone.0029872 (PMC3257241; doi:10.1371/journal.pone.0029872)
Supplement: Table S2 — Analyses of variants in the upstream region of CD1D in CCALD and AMN patients. (DOC) [file pone.0029872.s003.doc]

**Table S2**. Analyses of variants in the upstream region of *CD1D* in CCALD and AMN patients.

| SNP (dbSNP31) | MAF | MAF AMN | MAF CCALD | χ² | *P* value^a^ | empirical *P* value | Odds ratio [CI95%] |
| --- | --- | --- | --- | --- | --- | --- | --- |
| rs1570699 | 0.09 | 0.14 | 0.06 | 5.27 | 0.027 | 0.021 | 0.38 [0.16 ; 0.89] |
| rs10489821 | 0.09 | 0.13 | 0.06 | 4.22 | 0.045 | 0.057 | 0.41 [0.18 ; 0.98] |
| rs3087210 | 0.09 | 0.13 | 0.05 | 4.77 | 0.045 | 0.052 | 0.40 [0.17 ; 0.93] |
| rs3087211 | 0.09 | 0.13 | 0.05 | 4.77 | 0.045 | 0.052 | 0.40 [0.17 ; 0.93] |
| rs3087212 | 0.09 | 0.13 | 0.06 | 4.91 | 0.045 | 0.032 | 0.39 [0.17 ; 0.92] |
| rs10489823 | 0.09 | 0.13 | 0.06 | 4.77 | 0.045 | 0.041 | 0.40 [0.17 ; 0.93] |
| rs3021476 | 0.09 | 0.13 | 0.06 | 4.91 | 0.045 | 0.032 | 0.39 [0.17 ; 0.92] |

^a^: The Fisher's exact test was used for all SNPs.
